# The Effect of *Amanita rubescens* Pers Developmental Stages on Aroma Profile

**DOI:** 10.3390/jof7080611

**Published:** 2021-07-28

**Authors:** Jana Štefániková, Patrícia Martišová, Marek Šnirc, Vladimír Kunca, Július Árvay

**Affiliations:** 1 AgroBioTech Research Centre, Slovak University of Agriculture in Nitra, Tr. A. Hlinku 2, 949 76 Nitra, Slovakia; patricia.martisova@uniag.sk; 2 Department of Chemistry, Faculty of Biotechnology and Food Science, Slovak University of Agriculture in Nitra, Tr. A. Hlinku 2, 949 76 Nitra, Slovakia; marek.snirc@uniag.sk (M.Š.); julius.arvay@uniag.sk (J.Á.); 3 Department of Applied Ecology, Faculty of Ecology and Environmental Science, Technical University in Zvolen, Ul. T. G. Masaryka 24, 960 01 Zvolen, Slovakia; kunca@tuzvo.sk

**Keywords:** Blusher, caps, chromatography, mushrooms, stipes, volatile compounds

## Abstract

The dichloromethane extraction was applied to extracted volatile compounds of the six developmental stages of caps and stipes of an *Amanita rubescens* mushroom and the relative contents were measured with the gas chromatography-mass spectrometry. The number of identified compounds ranged between 53 and 52, respectively, with a high ratio of alkane volatiles. The significant differences between the aroma compounds were determined in caps to identify their stages of development. The fully mature stage caps were characterized by 4,6-dimethyl-dodecane (7.69 ± 1.15%), 2-hexyl-1-decanol (11.8 ± 1.61%), 1,3-di-tert-butylbenzene (11.4 ± 1.25%), heptadecyl pentadecafluorooctanoate (2.16 ± 0.31%), and 2-hexyl-1-dodecanol (13.5 ± 1.33%). Niacinamide (3.90 ± 0.07%) and glycerol (3.62 ± 1.27%) was present in the caps in the early-stage of the rotting mushroom, which represented the 10th–12th day of fructification. The caps and stipes from the 12th–15th day of fructification were characterized by 2,3-butanediol (11.7 ± 0.13% and 8.00 ± 0.10%, respectively). Moreover, the caps from this developmental stage were characterized by 2-methyl- and 3-methyl butanoic acids (0.18 ± 0.03% and 0.33 ± 0.02%, respectively) which are typical for the rotting stage. In this study, we confirmed the effect of *A. rubescens* developmental stages on the aroma profile.

## 1. Introduction

Aroma, formed by a combination of volatile and non-volatile compounds, is a key characteristic of food which significantly affects consumer preferences. Edible mushrooms are consumed as a delicacy not only because of their high nutritional value, but also because of their specific aroma [1]. Volatile organic compounds are synthesized as a protection of the organism or as by-products of metabolism [2]. They are released into the air and often have a characteristic odor [3]. There are many publications on fungal aroma. Altogether, 150 compounds have been identified, mainly from the categories of higher alcohols, ketones, esters, aldehydes, hydrocarbons, acids, heterocyclic and aromatic compounds [4,5,6,7,8]. In 1938, 1-octen-3-ol was first identified as a “mushroom–like flavor” and a “raw mushroom” with a characteristic earthy and sweet taste [5]. Other compounds have also been identified, specifically 1-octen-3-one, (E)-2-octen-1-ol, 1-octanol, 3-octanol, and (E)-2-octenal [9,10,11]. On the other hand, there is a lack of information around aroma profiles of different fruiting bodies and developmental stages of wild edible mushrooms, or *Amanita rubescens*.

European Blusher (*Amanita rubescens* Pers.) belongs to the *Amanitaceae* family. Many species of the family are inedible or toxic. On the other hand, *A. rubescens*, as well as some other species (*A. fulva, A. baccata, A. caesarea*), are edible [12,13,14,15]. The *A. rubescens* is a commonly collected and popular wild edible mushroom that is characterized by great sensory properties. Fruiting bodies of *A. rubescens* have a relatively high bioaccumulation capacity which can affect the spectrum of aromatic compounds and ultimately the increased risk of intoxication by heavy metals of the pickers [12].

According to SK Regulation 132/2014 [16] only wild edible mushrooms listed in the annex can be placed on the market. *A. rubescens* is absent from the annex, which contains 53 different wild edible mushroom species. In 2019, the consumption of “*other vegetables and mushrooms*” reached 13.3 kg per capita, according to Statistical Office of the Slovak Republic [17]. The consumption of exclusively wild edible mushrooms is not recorded in Slovakia. On the other hand, the Statistical Office of the Czech Republic recorded the consumption of 3.1 kg of “*mushrooms*” per capita in 2019 [18]. The aim of the work was to evaluate the effect of the various developmental stages of the *Amanita rubescens* Pers fruiting bodies from Slovakia on the volatile compounds, determined in a dichloromethane extract by the gas chromatography-mass spectrometry.

## 2. Materials and Methods

### 2.1. Sampling Area and Sampling

The six developmental stages of *A. rubescens* fruiting bodies (morphologically characterized by the expert mycologist prof. Kunca, and in compliance with the taxonomic keys [19]) were collected in Žakýlske Pleso area (Štavnické Vrchy, Slovakia) (GPS: 48°31′12.8″ N; 18°55′23.5″ E) on 3 July 2020. The area of the Štiavnické Vrchy is characterized by volcanic origins. The sampling area is predominantly forested with beech and hornbeam vegetation. The altitude of the sampling point is 757 m a.s.l., with an average annual temperature of 6–7 °C, and rainfall of 700–800 mm [20]. The region is characterized as very cold and humid, with a difference between potential evaporation and rainfall <50 mm (climate indicator for the months of June to August). The soil is characterized as loamy medium-heavy soil containing organic matter >20% [21].

**Figure 1:**
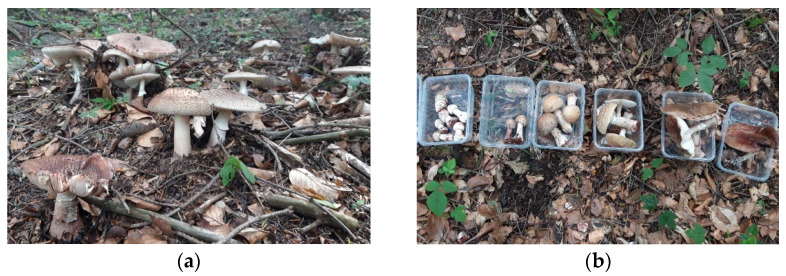
*Amanita rubescens* Pers: (**a**) Sampling place with different fruiting body developmental stages; (**b**) collected samples of six developmental stages in PE boxes.

The samples of individual developmental stages of *A. rubescens* fruiting bodies were taken in the morning from one 2 × 2 m area (Figure 1a). The advantage of such a collection is a high probability of homogeneous mycelium which creates a presumption of identical dynamics of nutrient uptake by the mycelium. Thus, identical or similar conditions for the formation of aromatic compounds were observed. After sampling, the fruiting bodies were removed from larger impurities and temporarily stored in ventilated polyethylene boxes (Figure 1b). Upon arrival at the laboratory, the samples were rinsed thoroughly in deionized water and divided into caps and stipes. The samples were dried at a temperature of 30 °C for ~22 h in a hot air dryer (Memmert UF 110m, Memmert GmbH & Co. KG, Schwabach, Germany). After thorough drying, the samples were homogenized on a rotary mill (IKA A10, IKA-Werke GmbH & Co. KG, Staufen, Germany) and stored in gas-tight 20 mL headspace vials.

**Figure 2:**
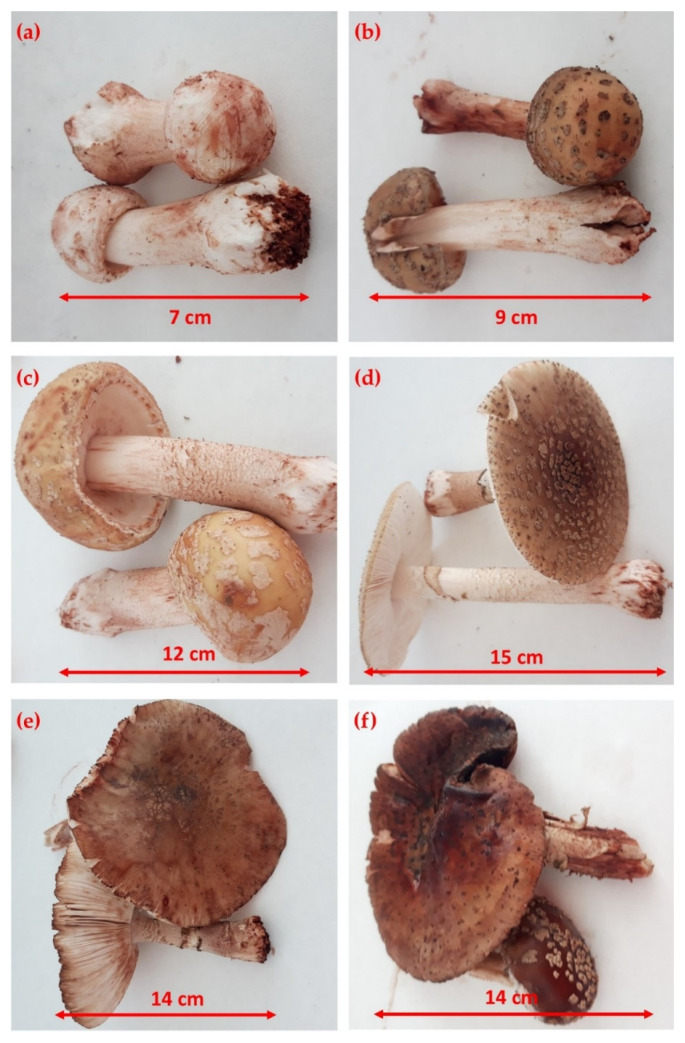
Different developmental stages of *Amanita rubescens* Pers: (**a**) 2nd–3rd day of fructification; (**b**) 3rd–5th day of fructification; (**c**) 5th–8th day of fructification; (**d**) 8th–10th day of fructification; (**e**) 10th–12th day of fructification; (**f**) 12th–15th day of fructification.

The specific shape and size of the six developmental stages of the mushroom *Amanita rubescens* Pers are shown in Figure 2. The six developmental stages were sorted, according to Falandysz et al. [22], by an expert mycologist (prof. Kunca). The first developmental stage (6 individuals) with the smallest fruiting bodies was estimated to be the 2nd–3rd day of fructification, with a size of 7 cm (Figure 2a). The 3rd to 5th day of fructification represented the second developmental stage (2 individuals), when the fruiting body reached 9 cm (Figure 2b). Figure 2c shows the third developmental stage (3 individuals), which was estimated to be the 5th–8th day of fructification, at a height of 12 cm of the mushroom body. The 8th–10th day stage (3 individuals), which is 15 cm high, is characterized as the fully mature stage of fructification and thus the fourth developmental stage (Figure 2d). Figure 2e shows the 5th developmental stage (the rotting stage) which was estimated to be the 10th–12th day of fructification, at a size of 14 cm (2 individuals). The sixth developmental stage (2 individuals) was estimated to be the 12th–15th day of fructification when the size of the fruiting body remained the same as the previous stage.

### 2.2. Extraction Technique

An amount of 0.3 g of the sample was extracted with dichloromethane for HPLC ≥99.8% (2.5 mL; Sigma-Aldrich Merck KGaA, Darmstadt, Germany) by a shaker (Unimax 2010, Heidolph, Schwabach, Germany) at laboratory temperature (18 °C ± 0.1 °C) for 4 h and then filtered through a syringe PVDF filter (0.22 µm × 13 mm; Chromservis, Bratislava, Slovakia). Afterward, 150 µL of the sample extracts were stored in 2 mL vials with the microvolume insert (Agilent Technologies Inc., Santa Clara, CA, USA).

### 2.3. Volatile Compounds Analysis

The analysis was carried out using gas chromatography-mass spectrometry (GC-MS) (GC 7890B coupled by MSD 5977A; Agilent Technologies Inc.) equipped with CombiPal autosampler CTC120 (CTC Analytics AG, Zwingen, Switzerland). A column HP-5ms (30 m × 0.25 mm × 0.25 µm; Agilent Technologies Inc.) was used. One microliter of the sample extract was injected in the inlet, operated in a split mode 10:1 at 250 °C. The oven temperature program started at 40 °C. The temperature was held for 3 min, then increased to 250 °C at 3 °C/min and then held again for 10 min. Helium was used as carrier gas at the constant flow (1.2 mL/min). The mass detector parameters were as follows: ionization energy of filament: 70 eV, transfer line temperature: 250 °C, MS source temperature: 230 °C, quadrupole temperature: 150 °C. The mass spectrometer was programmed under electron impact (EI) in a full-scan mode at m/z 40–450 with a frequency of 1.8 scans/s. Each sample was measured in triplicate.

### 2.4. Volatile Compounds Determination

The compound identification was carried out by comparing mass spectra (over 80% match) with a commercial database NIST library 2017 (National Institute of Standards and Technology, Gaithersburg, MD, USA) and Wiley library, retention times of the reference mixture standard of n-alkanes (11 components, Restek Corporation, Bellefonte, PA, USA), and a comparison of data on the occurrence of edible fungi with the literature [23,24]. The relative percentage (%) of the determined volatile compounds was calculated by dividing the individual peak area by the total area of all peaks. Peaks under 0.1% were not counted.

### 2.5. Statistical Analysis

All the data obtained were analyzed by descriptive statistics arithmetic average and standard deviation. Then, all the variables were tested for normality. According to the Shapiro-Wilk test and the Kolmogorov-Smirnov test, all the tested variables did not follow the Gaussian distribution. The Kruskal-Wallis test was performed to compare significant differences between the developmental stages. The ten most numerous volatile compounds, characteristic of each stage, were used for PCA analysis. Principal Component Analysis (Spearman type) was used to find a pattern of similarity of the observations and the variables by displaying them as points on a map. Descriptive statistics, normality tests, Kruskal-Wallis test, and the PCA analysis were performed using the MS Excel and XLSTAT package program [25].

## 3. Results

In total, the sixty-two volatile compounds were identified by GC-MS analysis in dichloromethane extracts of the mushroom *A. rubescens* Pers. including 24 alkanes, 13 alcohols, 11 alkenes, 5 acids, 3 esters, 2 aldehydes, 2 amides, 1 imide, and 1 ketone. The values shown in Table 1 and Table 2 for each volatile compound are means of triplicate determinations with standard deviation. The developmental stages of *A. rubescens* were sorted into six developmental stages and the evaluation of the aroma composition in caps and stipes of the fruiting bodies were determined independently.

The 2-hexyl-1-decanol (9.26–15.0% and 10.3–16.8%), 2-hexyl-1-dodecanol (13.2–16.2% and 6.00–15.3%), 2-butyl-1-octanol (3.95–6.58% and 4.91–6.19%), 2,4-di-tert-butylphenol (8.31–14.2% and 5.85–11.3%), 1,3-di-tert-butylbenzene (8.96–13.0% and 9.97–12.4%), 4,6-dimethyl-dodecane (6.40–8.72% and 6.35–8.88%), and 2,6-dimethyl-nonane (2.32–4.21% and 3.09–3.92%) in caps and stipes of *A. rubescens* showed high relative percentages. The caps contained more volatile compounds than stipes with different relative content. The relative percentage of 2,4-dimethyl-1-heptene in caps was 3.65–5.81% for developmental stages 1–5 (it was one of the higher contents) and 1.46% for developmental stage 6, while the relative percentage for stipes were 1.95–4.96%, and 3.75%, respectively.

Several unique volatiles that were present in one developmental stage and not in others can be marked as markers. The cap in stage 1 had the unique volatile succinimide. The cap in the next developmental stage 2 had the unique volatiles 7-methyl-6-tridecene and 5-ethyl-5-methyl-decane. The cap in stage 3 had no specific marker. The fully mature stage had the unique volatile 2-octyl-1-dodecanol. The early-stage of the rotting mushroom had specific volatiles N-methyliminopropylbenzene and nonanoic acid. The cap in developmental stage 6 had several unique acids: 2-methyl-butanoic acid and 3-methyl-butanoic acid.

The differences were also recorded in stipes. There were no markers determined in developmental stages 1, 3, and 4. The stipes in stage 2 were characterized by 11-methyldodecanol, 3-methoxy-1-butanol, 1,2-diethyl-cyclooctane, 1-iodo-eicosane, and tetradecyl-pentadecafluorooctanoate. The stipe of the early-stage of the rotting was characterized by 1-iodo-dodecane and the latest developmental stage included 3-methoxy-1-butanol acetate, α-bulnesene, 2,3-butanediol, dodecanoic acid, and tetradecanoic acid.

Moreover, different fructification stages (unripe and overripe) can be distinguished based on the presence of volatiles in the caps. The first two stages (1st and 2nd), which can be marked as the unripe stage, contained 4-methyl-dodecan-1-ol, 2-hydroxy-2,N-dimethyl-butanamide, 1,3,5-trimethyl-cyclohexane, 4-methylene-decane, heptadecane, 5-methyl-tridecane, 4,6-dimethyl-undecane, and 5-methyl-undecane. For the overripe or rotting mushroom stages (5th and 6th stage) compounds 2,3-butanediol and glycerol were identified.

**Figure 3:**
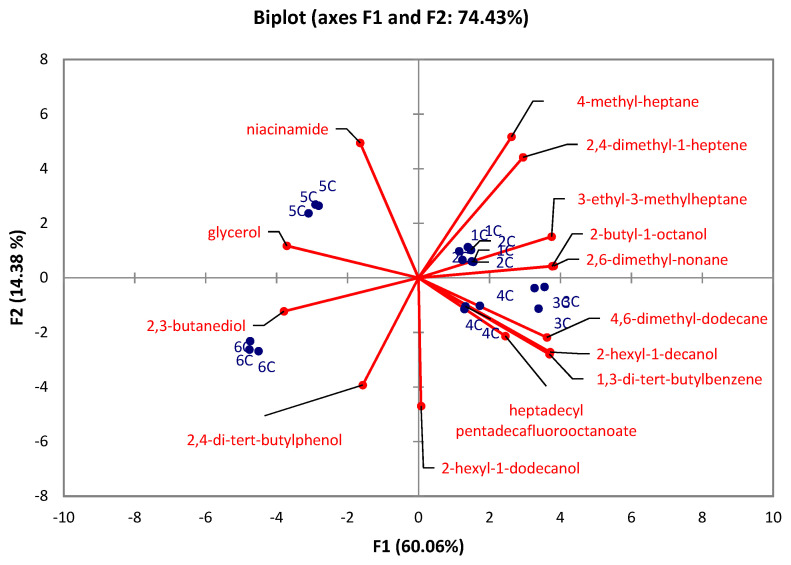
PCA evaluation of six developmental stages (1C–6C) of *A. rubescens* Pers caps.

**Figure 4:**
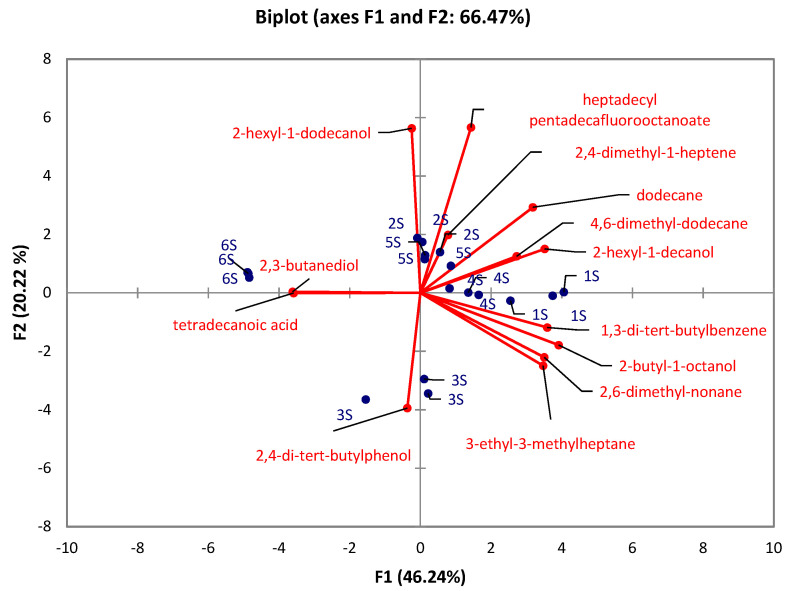
PCA evaluation of six developmental stages (1S–6S) of *A. rubescens* Pers stipes.

There were significant differences (Kruskal-Wallis test) observed in the caps between the developmental stages in all identified compounds (*p* < 0.05) except for 7-methyl-6-tridecene (*p* = 0.1176), and 5-ethyl-5-methyl-decane (*p* = 0.1176). At the same time, there were no significant differences in stipes between the developmental stages in 1-tetradecene (*p* = 0.1215), 2,5-dimethyl-2,5-hexanediol (*p* = 0.0568), 2,6,10-trimethyltridecane (*p* = 0.6627), 3,7,11,15-tetramethyl-2-hexadecene (*p* = 0.0639), 1-iodo-dodecane (*p* = 0.1176), tetradecyl pentadecafluorooctanoate (*p* = 0.1176), pentadecane (*p* = 0.1489), 3,8-dimethyl-undecane (*p* = 0.0793), 4,6-dimethyl-undecane (*p* = 0.2261), and 4,8-dimethyl-undecane (*p* = 0.0759). The PCA for caps (Figure 3) revealed that 74.43% of the total variation embodied in 14 variables could be effectively condensed and explained by the first two principal components (PCs), with eigenvalues of 8.4 and 2.01, respectively. The PCA for stipes (Figure 4) explains 66.47% of total variability with the eigenvalues of the first two principal components (PCs) 6.01 and 2.63, respectively. The results show that stage 6 in both PCAs (caps, stipes) is characterized by 2,3-butanediol. In the case of caps, the early-stage of the rotting mushroom (stage 5) can be characterized by glycerol and niacinamide contents. In the case of stipes, it is possible to strictly characterize stage 3 by 2,4-di-tert-butylphenol. Other stages cannot be clearly distinguished.

## 4. Discussion

In this study, the fruit bodies of *A. rubescens* were divided into six developmental stages. According to Kalač [30], the lifetime of the fruiting body is estimated to be only 10–14 days. In this study, the sixth developmental stage was estimated to be the 12th–15th day of fructification, characterized by a dark brown color. The results showed that the caps contained higher quantities of volatile compounds than the stipes. On the other hand, the rotting mushroom cap stages 5 and 6 were statistically (PCA and Kruskal-Wallis test) characterized by unique aroma markers (2,3-butanediol (*p* < 0.0001), glycerol (*p* < 0.0001), and niacinamide (*p* = 0.0068)). The 2-methyl and 3-methyl butanoic acids (*p* = 0.0074) were characterized only for the 6th developmental stage and according to the literature, it is known to be sweaty [28]. In general, the acid compounds (i.e., nonanoic acid, and 2-methyl- and 3-methyl- butanoic acids) are responsible for the sweaty attribute, which is unpleasant and had a negative effect on the aroma profile of the mushrooms [31,32,33]. Most publications have focused on the sensory aroma quality of mushrooms from a technological aspect [11,34,35,36,37], while very few publications examined the sensory properties of mushroom developmental stages [38]. Lu et al. [38] characterized the aroma profile of two truffle species (*T. indicum* and *T. pseudohimalayense*) and the influence of the maturation stage on volatile organic compounds. The unripe stage was characterized by volatile compounds from groups: alcohols and phenols (7), esters (9), aldehydes and ketones (6), hydrocarbons (3), and 1 *N*-containing compound. In the mature stage, volatile compounds such as esters (2), alcohol (1), aldehydes and ketones (4), benzenes and methoxy compounds (5), hydrocarbons (2), and *N*-containing compounds (2) were identified. The same compound groups were also identified in the dichloromethane extracts of *Amanita rubescens* in our study.

According to the literature, the 1-octen-3-one is characteristic of a metallic mushroom-like odor with a low-odor threshold, and it makes a bigger contribution to the odor impressions of mushrooms than 1-octen-3-ol, which is known to have a higher content [8]. In this study, a series of eight-carbon aliphatic components were not detected in dichloromethane extracts of *A. rubescens* except 2-butyl-octanol (3.95–6.58%). Murray et al. [39] determined the key odorants responsible for the unique aroma of the diethyl ether extract of fragrant bolete, *Suillus punctipes*. The compounds 1-octen-3-one, 1-octen-3-ol, (2E)-oct-2-enal, linalool, δ-dodecalactone, and a mixture of octanal, nonanal, and decanal were essential for the unique aroma profile of *S. punctipes*. The n-hexadecanoic acid; 9,12-octadecadienoic acid (Z,Z)-, and 2(3H)-furanone were the most frequently occurring compounds in the methanolic extract of the *Lentinus squarrosulus*, *Auricularia auricula-judae*, *Mycetinis copelandii*, *Baeospora myosura*, *Pleurotus ostreatus*, and *Volvariella volvacea* [40]. De Pinho et al. [41] used the headspace solid-phase microextraction technique (HS-SPME) in the headspace of a wild edible mushroom powder and in the headspace of a mushroom dissolved in 10% ethanol. Their study included *A. rubescens* aromatic profile and it was characterized by 3-octanone, 3-octanol, benzoic acid, undecanal, and α-pinene. According to Portalo-Calero et al. [23] the 3-methyl-butanal, 2-methyl-butanal, hexanal, styrene, 3-octenol, 3-octanone, 3-octanol, and 2-ethyl-hexanol were identified by HS-SPME GC-MS in *A. rubescens*. This study is a first step in aiming to advance an understanding of the differentiation of developmental stages of *Amanita rubescens* through dichloromethane extracts.

## 5. Conclusions

This study provided a thorough comparison of the volatile compound profiles of *Amanita rubescens* at various developmental stages of fruiting bodies. Our results for the volatile compounds in *A. rubescens* show a minor change for caps and stipes during the fruit body maturation (from the smallest fruit body to full maturity). On the other hand, the eight compounds (4-methyl-dodecan-1-ol, 2-hydroxy-2,N-dimethyl-butanamide, 1,3,5-trimethy-cyclohexane, 4-methylene-decane, heptadecane, 5-methyl-tridecane, 4,6-dimethyl-undecane, and 5-methyl-undecane) were identified in the earlier stages of the fruit body while they were absent at the full-maturity stage. Statistical analysis showed that several compounds displayed different relationship patterns in the cap the rotting mushroom stage (2,3-butanediol, glycerol, and niacinamide) from that of the full-maturity stage (4,6-dimethyl-dodecane, 2-hexyl-1-decanol, 1,3-di-tert-butylbenzene, heptadecyl pentadecafluorooctanoate, and 2-hexyl-1-dodecanol). We confirmed that, based on aromatic profiles, it is possible to distinguish the initial developmental stage of a fruiting body from the fully mature and the decomposition stage. At the same time, it is better to determine the aromatic profiles of mushrooms in caps than stipes. For the first time, this study specifies the individual developmental stages of the wild edible mushroom *Amanita rubescens*. In conclusion, there is still a need for further confirmation of volatile organic compounds on more individual fruiting bodies. We recommend studying developmental stages of *A. rubescens* from different localities in Slovakia (possible effect of chemical-physical, and geochemical parameters of soil) or comparing the results with different developmental stages of other wild edible mushrooms.

## Figures and Tables

**Table 1:** Relative percentage (%) of volatile compounds identified in the dichloromethane extract of the *Amanita rubescens* caps (1–6C) ^1^ in various developmental stages by the gas chromatography-mass spectrometry.

#	RT (min)	Compound	Sensors ^2^	1C	2C	3C	4C	5C	6C	Kruskal-Wallis Test (*p*-Values)
				Peak Area (%) ± SD ^3^						
Acids (4)										
1	6.37	2-methyl-butanoic acid	Cashew, Cheese, Fruity, Overripe, Sweaty, Sweet	ND ^4^	ND	ND	ND	ND	0.18 ± 0.03	0.0074
2	6.02	3-methyl-butanoic acid	Acidic, Cheese, Rancid, Sweaty	ND	ND	ND	ND	ND	0.33 ± 0.02	0.0074
3	25.37	nonanoic acid	Fatty, Green, Musty, Sour, Sweaty, Waxy	ND	ND	ND	ND	0.82 ± 0.14	ND	0.0074
4	44.69	tetradecanoic acid	Fatty, Nearly odorless, Soupy, Very faint, Waxy	ND	ND	ND	1.11 ± 0.92	1.80 ± 0.14	ND	0.0006
Alcohols (11)										
5	11.20	glycerol	Odorless	ND	ND	ND	ND	3.62 ± 1.27	1.26 ± 0.51	<0.0001
6	28.12	2-hexyl-1-decanol	Not found	11.6 ± 0.19	11.6 ± 0.33	15.0 ± 0.21	11.8 ± 1.61	9.26 ± 0.16	10.2 ± 0.09	<0.0001
7	40.69	2-hexyl- 1-dodecanol	Not found	14.6 ± 0.63	14.4 ± 0.46	15.0 ± 0.91	13.5 ± 1.33	13.2 ± 0.91	16.2 ± 1.09	0.0005
8	46.00	2-octyl- 1-dodecanol	Not found	ND	ND	ND	1.14 ± 0.06	ND	ND	0.0074
9	16.46	2-butyl-1-octanol	Not found	5.70 ± 0.04	5.88 ± 0.09	6.58 ± 0.06	5.50 ± 0.59	4.69 ± 0.09	3.95 ± 0.08	<0.0001
10	4.28	2,3-butanediol	Fruity, Onion	ND	ND	ND	ND	0.65 ± 0.01	11.7 ± 0.13	<0.0001
11	35.44	2,4-di-tert-butylphenol	Not found	8.31 ± 0.04	8.73 ± 0.30	13.7 ± 0.54	10.2 ± 1.34	12.5 ± 0.67	14.2 ± 0.66	<0.0001
12	15.27	2,5-dimethyl-2,5-hexanediol	Not found	0.74 ± 0.02	0.73 ± 0.01	0.89 ± 0.02	0.74 ± 0.08	0.59 ± 0.08	0.55 ± 0.09	0.0005
13	10.84	tetrahydro-2,5-dimethyl-2H-pyranmethanol	Not found	0.99 ± 0.02	0.96 ± 0.05	1.08 ± 0.01	0.81 ± 0.04	0.75 ± 0.03	0.39 ± 0.02	<0.0001
14	14.12	4-methyl-dodecan-1-ol	Not found	0.20 ± 0.00	0.19 ± 0.02	ND	ND	ND	ND	0.0006
15	14.04	benzyl alcohol	Aromatic, Floral, Fruity, Phenolic, Rose, Sweat, Sweet	0.57 ± 0.01	0.50 ± 0.02	ND	0.44 ± 0.01	0.57 ± 0.08	0.76 ± 0.07	<0.0001
Aldehydes (2)										
16	10.52	benzaldehyde	Almond, Burnt sugar, Fruity, Powdery, Woody	0.17 ± 0.01	0.16 ± 0.02	ND	0.12 ± 0.01	ND	ND	<0.0001
17	17.48	nonanal	Aldehydic, Floral, Fruity, Fatty, Green, Rose, Sweet, Tallowy, Waxy	0.20 ± 0.01	ND	ND	0.14 ± 0.03	ND	ND	0.0011
Alkanes (21)										
18	32.63	2,6,10-trimethyltridecane	Not found	0.41 ± 0.01	0.43 ± 0.09	ND	0.42 ± 0.05	0.41 ± 0.02	0.29 ± 0.01	0.0076
19	12.92	3-ethyl-3-methylheptane	Not found	2.95 ± 0.03	2.97 ± 0.07	3.43 ± 0.04	2.79 ± 0.26	2.42 ± 0.04	1.79 ± 0.06	<0.0001
20	6.27	1,3,5-trimethyl-cyclohexane	Not found	0.14 ± 0.01	0.13 ± 0.01	ND	ND	ND	ND	0.0008
21	12.50	decane	Alkane, Fruity, Fusel, Sweet	0.66 ± 0.02	0.65 ± 0.01	0.75 ± 0.01	0.64 ± 0.05	0.60 ± 0.04	0.34 ± 0.08	0.0004
22	26.29	5-ethyl-5-methyl-decane	Not found	ND	0.18 ± 0.03	ND	ND	ND	ND	0.1176
23	19.06	dodecane	Alkane, Fusel	1.59 ± 0.04	1.51 ± 0.03	1.46 ± 0.01	1.51 ± 0.24	1.31 ± 0.07	1.21 ± 0.04	0.0044
24	30.95	4,6-dimethyl-dodecane	Not found	7.88 ± 0.15	7.59 ± 0.36	8.72 ± 0.53	7.69 ± 1.15	6.40 ± 0.59	6.59 ± 0.17	<0.0001
25	43.09	eicosane	Alkane, Fruity, Sweet, Woody	0.72 ± 0.07	0.56 ± 0.09	ND	0.57 ± 0.14	0.42 ± 0.13	0.33 ± 0.03	0.0006
26	42.48	heptadecane	Alkane, Fusel	0.31 ± 0.13	0.39 ± 0.01	ND	ND	ND	ND	0.0005
27	5.21	2,4-dimethyl-heptane	Not found	0.59 ± 0.01	0.57 ± 0.03	ND	0.40 ± 0.03	0.49 ± 0.03	0.18 ± 0.01	<0.0001
28	3.74	4-methyl-heptane	Not found	2.01 ± 0.02	1.84 ± 0.03	1.96 ± 0.02	1.06 ± 0.14	1.63 ± 0.03	0.30 ± 0.03	<0.0001
29	38.81	hexadecane	Alkane, Fruity, Fusel, Sweet	0.34 ± 0.00	0.46 ± 0.05	ND	0.40 ± 0.05	0.30 ± 0.08	0.37 ± 0.03	0.0006
30	41.73	2,6,10,14-tetramethyl-hexadecane	Not found	1.21 ± 0.03	1.05 ± 0.06	ND	1.25 ± 0.36	0.42 ± 0.03	0.49 ± 0.05	<0.0001
31	13.11	2,6-dimethyl-nonane	Not found	3.64 ± 0.03	3.66 ± 0.06	4.21 ± 0.05	3.46 ± 0.34	3.07 ± 0.11	2.32 ± 0.07	<0.0001
32	10.72	4-methyl-nonane	Not found	0.42 ± 0.02	0.43 ± 0.00	ND	0.38 ± 0.05	0.34 ± 0.01	0.23 ± 0.02	<0.0001
33	30.32	pentadecane	Alkane, Fusel, Mild green	0.70 ± 0.02	0.67 ± 0.12	ND	0.64 ± 0.12	0.61 ± 0.11	0.29 ± 0.02	0.0023
34	30.87	tetradecane	Alkane, Fusel, Mild herbaceous, Sweet	1.46 ± 0.01	1.5 ± 0.03	1.73 ± 0.08	1.32 ± 0.19	1.13 ± 0.08	1.25 ± 0.03	<0.0001
35	28.89	5-methyl-tridecane	Not found	0.13 ± 0.01	0.17 ± 0.06	ND	ND	ND	ND	0.0002
36	22.10	4,6-dimethyl-undecane	Not found	0.15 ± 0.02	0.30 ± 0.06	ND	ND	ND	ND	<0.0001
37	22.69	4,8-dimethyl-undecane	Not found	0.34 ± 0.06	0.41 ± 0.11	ND	0.36 ± 0.06	0.39 ± 0.06	0.26 ± 0.01	0.0044
38	19.94	5-methyl-undecane	Not found	0.17 ± 0.01	0.17 ± 0.04	ND	ND	ND	ND	0.0053
Alkenes (10)										
39	25.87	1-tetradecene	Not found	0.83 ± 0.04	0.62 ± 0.04	ND	0.66 ± 0.06	0.52 ± 0.07	0.59 ± 0.02	0.0003
40	5.85	2,4-dimethyl-1-heptene	Not found	5.51 ± 0.03	5.31 ± 0.12	5.81 ± 0.05	3.65 ± 0.20	4.49 ± 0.10	1.46 ± 0.02	<0.0001
41	24.20	3,7,11,15-tetramethyl-2-hexadecene	Not found	0.45 ± 0.03	0.40 ± 0.03	ND	0.44 ± 0.04	0.37 ± 0.07	0.40 ± 0.04	0.0074
42	13.47	7-methyl-4-decene	Not found	0.76 ± 0.01	0.77 ± 0.02	0.85 ± 0.02	0.70 ± 0.06	0.63 ± 0.06	0.47 ± 0.05	<0.0001
43	25.54	7-methyl-6-tridecene	Not found	ND	0.14 ± 0.01	ND	ND	ND	ND	0.1176
44	24.41	1,3-di-tert-butylbenzene	Not found	11.2 ± 0.17	11.4 ± 0.16	13.0 ± 0.09	11.4 ± 1.25	8.96 ± 0.29	9.48 ± 0.05	<0.0001
45	14.60	4-methylene-decane	Not found	0.13 ± 0.00	0.12 ± 0.02	ND	ND	ND	ND	0.0007
46	33.94	heneicosane	Alkane, Odorless	0.68 ± 0.04	0.62 ± 0.06	ND	0.64 ± 0.18	ND	0.47 ± 0.04	0.0009
47	20.77	n-methyliminopropylbenzene	Not found	ND	ND	ND	ND	0.50 ± 0.04	ND	0.0074
48	4.80	tetrachloroethylene	Chloroform	0.12 ± 0.00	0.13 ± 0.01	ND	0.14 ± 0.02	ND	ND	0.0022
Amides (2)										
49	8.19	2-hydroxy-2,N-dimethyl-butanamide	Not found	0.63 ± 0.02	0.64 ± 0.02	ND	ND	ND	ND	0.0006
50	30.26	niacinamide	Not found	0.64 ± 0.08	0.91 ± 0.07	1.94 ± 0.01	0.63 ± 0.76	3.90 ± 0.07	0.82 ± 0.05	0.0068
Ester (1)										
51	45.03	heptadecyl pentadecafluorooctanoate	Not found	1.99 ± 0.45	2.20 ± 0.05	2.20 ± 0.11	2.16 ± 0.31	1.83 ± 0.38	1.86 ± 0.12	0.0238
Imide (1)										
52	18.58	succinimide	Not found	0.16 ± 0.00	ND	ND	ND	ND	ND	0.0074
Ketone (1)										
53	19.12	2,3-dihydro-3,5-dihydroxy-6-methyl-4H-pyran-4-one	Not found	ND	0.17 ± 0.02	ND	0.39 ± 0.41	0.68 ± 0.08	ND	<0.0001

^1^ 1C—the first developmental stage of caps; 2C—the second developmental stage of caps; 3C—the third developmental stage of caps; 4C—the fourth developmental stage of caps; 5C—the fifth developmental stage of caps; 6C—the sixth developmental stage of caps. ^2^ Sensory descriptors from AroChemBase database (Alpha M.O.S., Toulouse, France), The Good Scents Company Information System [26], Selli et al. [27], Zhang et al. [28], Zhuang et al. [29]. ^3^ SD: standard deviation, values represent means of three replicate determinations. ^4^ ND: not detected.

**Table 2:** Relative percentage (%) of volatile compounds identified in the dichloromethane extract of the *Amanita rubescens* stipes (1–6S) ^1^ at various developmental stages by the gas chromatography-mass spectrometry.

#	RT (min)	Compound	Sensors ^2^	1S	2S	3S	4S	5S	6S	Kruska-Wallis Test (*p*-Values)
				Peak Area (%) ± SD ^3^						
Acids (2)										
1	37.44	dodecanoic acid	Fatty	ND ^4^	ND	ND	ND	ND	0.46 ± 0.15	0.0074
2	44.69	tetradecanoic acid	Fatty, Nearly odorless, Soupy, Very faint, Waxy	ND	ND	ND	ND	ND	11.4 ± 0.78	0.0074
Alcohols (11)										
3	34.08	11-methyldodecanol	Not found	ND	0.45 ± 0.10	ND	ND	ND	ND	0.0074
4	8.19	3-methoxy-1-butanol	Not found	ND	0.66 ± 0.03	ND	ND	ND	ND	0.0074
5	28.12	2-hexyl-1-decanol	Not found	16.8 ± 3.46	12.8 ± 0.30	11.4 ± 2.46	12.7 ± 0.20	13.4 ± 0.41	10.3 ± 0.07	0.0037
6	40.69	2-hexyl-1-dodecanol	Not found	7.80 ± 0.17	15.3 ± 1.25	6.00 ± 0.17	13.8 ± 0.32	14.9 ± 0.66	10.6 ± 0.44	<0.0001
7	16.46	2-butyl-1-octanol	Not found	6.19 ± 0.09	5.47 ± 0.16	5.61 ± 0.18	5.83 ± 0.12	5.57 ± 0.17	4.91 ± 0.06	0.0002
8	4.28	2,3-butanediol	Fruity, Onion	ND	ND	ND	ND	ND	8.00 ± 0.10	0.0074
9	35.44	2,4-di-tert-butylphenol	Not found	5.85 ± 0.33	6.27 ± 0.23	11.3 ± 0.28	9.39 ± 0.11	9.25 ± 0.08	6.51 ± 0.28	<0.0001
10	15.27	2,5-dimethyl-2,5-hexanediol	Not found	0.76 ± 0.01	0.75 ± 0.06	0.76 ± 0.03	0.78 ± 0.02	0.71 ± 0.07	0.65 ± 0.04	0.0568
11	10.84	tetrahydro-2,5-dimethyl-2H-pyranmethanol	Not found	0.93 ± 0.01	1.00 ± 0.01	0.71 ± 0.03	0.81 ± 0.03	0.64 ± 0.02	0.79 ± 0.00	<0.0001
12	14.12	4-methyl-dodecan-1-ol	Not found	0.20 ± 0.02	0.17 ± 0.02	0.16 ± 0.03	0.18 ± 0.03	0.16 ± 0.02	ND	0.0271
13	14.04	benzyl alcohol	Aromatic, Floral, Fruity, Phenolic, Rose, Sweat, Sweet	0.45 ± 0.02	0.38 ± 0.05	0.43 ± 0.02	0.44 ± 0.01	0.41 ± 0.01	0.42 ± 0.02	0.0167
Aldehyde (1)										
14	10.52	benzaldehyde	Almond, Burnt sugar, Fruity, Powdery, Woody	0.11 ± 0.09	0.15 ± 0.03	ND	0.13 ± 0.00	0.08 ± 0.07	ND	0.0316
Alkanes (23)										
15	32.63	2,6,10-trimethyltridecane	Not found	0.49 ± 0.06	0.48 ± 0.11	0.31 ± 0.27	0.45 ± 0.10	0.5 ± 0.07	0.44 ± 0.02	0.6627
16	12.92	3-ethyl-3-methylheptane	Not found	3.22 ± 0.05	2.68 ± 0.07	2.81 ± 0.09	2.94 ± 0.08	2.58 ± 0.08	2.51 ± 0.02	<0.0001
17	25.40	1,2-diethyl-cyclooctane	Not found	ND	0.19 ± 0.05	ND	ND	ND	ND	0.0074
18	12.50	decane	Alkane, Fruity, Fusel, Sweet	0.71 ± 0.02	0.62 ± 0.02	0.42 ± 0.36	0.65 ± 0.03	0.56 ± 0.02	0.55 ± 0.01	0.0026
19	19.06	dodecane	Alkane, Fusel	1.82 ± 0.09	1.55 ± 0.09	1.31 ± 0.06	1.63 ± 0.08	1.70 ± 0.05	1.39 ± 0.04	<0.0001
20	26.04	1-iodo-dodecane	Not found	ND	ND	ND	ND	0.1 ± 0.09	ND	0.1176
21	30.95	4,6-dimethyl-dodecane	Not found	8.56 ± 0.30	8.41 ± 0.66	8.40 ± 0.78	8.43 ± 0.24	8.88 ± 0.37	6.35 ± 0.19	0.0332
22	43.09	eicosane	Alkane, Fruity, Sweet, Woody	0.66 ± 0.06	1.00 ± 0.02	0.63 ± 0.10	0.62 ± 0.06	1.27 ± 0.30	0.55 ± 0.06	0.0024
23	43.49	1-iodo-eicosane	Not found	ND	0.82 ± 0.07	ND	ND	ND	ND	0.0074
24	42.48	heptadecane	Alkane, Fusel	0.35 ± 0.01	0.30 ± 0.15	0.39 ± 0.04	0.42 ± 0.00	0.41 ± 0.06	0.23 ± 0.02	0.0173
25	5.21	2,4-dimethyl-heptane	Not found	0.52 ± 0.01	0.48 ± 0.01	0.32 ± 0.02	0.39 ± 0.01	0.24 ± 0.01	0.40 ± 0.01	<0.0001
26	3.74	4-methyl-heptane	Not found	1.49 ± 0.03	1.50 ± 0.04	0.70 ± 0.02	0.97 ± 0.07	0.45 ± 0.03	1.14 ± 0.03	<0.0001
27	38.81	hexadecane	Alkane, Fruity, Fusel, Sweet	0.67 ± 0.05	0.45 ± 0.05	0.29 ± 0.25	0.41 ± 0.03	0.48 ± 0.03	0.38 ± 0.04	0.0030
28	41.73	2,6,10,14-tetramethyl-hexadecane	Not found	1.14 ± 0.03	1.69 ± 0.10	1.13 ± 0.17	1.36 ± 0.19	1.27 ± 0.17	0.79 ± 0.14	0.0019
29	13.11	2,6-dimethyl-nonane	Not found	3.92 ± 0.05	3.43 ± 0.04	3.51 ± 0.07	3.65 ± 0.03	3.18 ± 0.07	3.09 ± 0.03	<0.0001
30	10.72	4-methyl-nonane	Not found	0.45 ± 0.03	0.38 ± 0.02	0.38 ± 0.01	0.4 ± 0.03	0.32 ± 0.03	0.35 ± 0.01	0.0001
31	30.32	pentadecane	Alkane, Fusel, Mild green	0.74 ± 0.13	0.74 ± 0.10	0.65 ± 0.23	0.70 ± 0.05	0.76 ± 0.14	0.56 ± 0.11	0.1489
32	30.87	tetradecane	Alkane, Fusel, Mild herbaceous, Sweet	1.90 ± 0.03	1.63 ± 0.07	0.97 ± 0.84	1.45 ± 0.04	1.72 ± 0.07	1.31 ± 0.03	<0.0001
33	28.89	5-methyl-tridecane	Not found	0.17 ± 0.03	0.19 ± 0.00	0.15 ± 0.03	0.11 ± 0.09	0.17 ± 0.02	0.14 ± 0.00	0.0362
34	26.29	3,8-dimethyl-undecane	Not found	0.18 ± 0.03	0.10 ± 0.09	0.24 ± 0.12	0.19 ± 0.02	0.17 ± 0.15	ND	0.0793
35	22.10	4,6-dimethyl-undecane	Not found	0.31 ± 0.04	0.30 ± 0.04	0.24 ± 0.21	0.33 ± 0.04	0.35 ± 0.07	0.13 ± 0.02	0.2261
36	22.69	4,8-dimethyl-undecane	Not found	0.41 ± 0.07	0.28 ± 0.03	0.31 ± 0.05	0.35 ± 0.06	0.40 ± 0.14	0.29 ± 0.02	0.0759
37	19.94	5-methyl-undecane	Not found	0.18 ± 0.05	0.19 ± 0.01	ND	ND	0.21 ± 0.01	ND	0.0001
Alkenes (9)										
38	25.87	1-tetradecene	Not found	0.68 ± 0.02	0.76 ± 0.15	0.66 ± 0.05	0.68 ± 0.05	0.68 ± 0.04	0.55 ± 0.06	0.1215
39	5.85	2,4-dimethyl-1-heptene	Not found	4.96 ± 0.07	4.69 ± 0.08	1.95 ± 1.69	3.54 ± 0.06	2.12 ± 0.05	3.75 ± 0.03	<0.0001
40	24.20	3,7,11,15-tetramethyl-2-hexadecene	Not found	0.44 ± 0.03	0.39 ± 0.04	0.27 ± 0.24	0.45 ± 0.03	0.44 ± 0.02	0.38 ± 0.06	0.0639
41	13.47	7-methyl-4-decene	Not found	0.82 ± 0.01	0.71 ± 0.02	0.72 ± 0.02	0.73 ± 0.02	0.67 ± 0.01	0.62 ± 0.02	<0.0001
42	35.27	α-bulnesene	Not found	ND	ND	ND	ND	ND	0.39 ± 0.03	0.0074
43	24.41	1,3-di-tert-butylbenzene	Not found	12.3 ± 0.19	11.7 ± 0.12	12.1 ± 0.24	12.1 ± 0.20	12.4 ± 0.19	9.97 ± 0.14	0.0026
44	14.60	4-methylene-decane	Not found	0.14 ± 0.01	0.08 ± 0.07	ND	0.13 ± 0.01	ND	0.14 ± 0.01	0.0003
45	33.94	heneicosane	Waxy	0.57 ± 0.15	0.86 ± 0.27	0.65 ± 0.11	0.57 ± 0.12	0.72 ± 0.10	0.12 ± 0.10	0.0004
46	4.80	tetrachloroethylene	Not found	0.16 ± 0.02	ND	0.16 ± 0.01	0.14 ± 0.01	ND	ND	0.0001
Amides (2)										
47	8.19	2-hydroxy-2,N-dimethyl-butanamide	Not found	0.57 ± 0.05	ND	ND	0.46 ± 0.03	ND	ND	<0.0001
48	30.26	niacinamide	Not found	0.09 ± 0.08	ND	0.24 ± 0.27	0.22 ± 0.05	0.26 ± 0.03	0.58 ± 0.10	0.0035
Esters (3)										
49	45.03	heptadecyl pentadecafluorooctanoate	Not found	2.30 ± 0.07	2.63 ± 0.34	ND	2.32 ± 0.05	2.58 ± 0.06	1.99 ± 0.05	<0.0001
50	25.08	tetradecyl pentadecafluorooctanoate	Not found	ND	0.14 ± 0.13	ND	ND	ND	ND	0.1176
51	8.207	3-methoxybutyl acetate	Not found	ND	ND	ND	ND	ND	0.48 ± 0.02	0.0074
Ketone (1)										
52	19.12	2,3-dihydro-3,5-dihydroxy-6-methyl-4H-pyran-4-one	Not found	ND	ND	0.15 ± 0.13	0.15 ± 0.02	ND	0.24 ± 0.05	0.0024

^1^ 1S—the first developmental stage of stipes; 2S—the second developmental stage of stipes; 3S—the third developmental stage of stipes; 4S—the fourth developmental stage of stipes; 5S—the fifth developmental stage of stipes; 6S—the sixth developmental stage of stipes. ^2^ Sensory descriptors from AroChemBase database (Alpha M.O.S., Toulouse, France), The Good Scents Company Information System [26], Selli et al. [27], Zhang et al. [28], Zhuang et al. [29]. ^3^ SD: standard deviation, values represent means of three replicate determinations. ^4^ ND: not detected.
